# Extracting Semantics from Question-Answering Services for Snippet Reuse

**DOI:** 10.1007/978-3-030-45234-6_6

**Published:** 2020-03-13

**Authors:** Themistoklis Diamantopoulos, Nikolaos Oikonomou, Andreas Symeonidis

**Affiliations:** 8 University of Paderborn, Paderborn, Germany; 9 ICREA, Open University of Catalonia, Barcelona, Spain; Electrical and Computer Engineering Department, Aristotle University of Thessaloniki, Thessaloniki, Greece

**Keywords:** Code Search, Snippet Mining, Code Semantic Analysis, Question-Answering Systems

## Abstract

Nowadays, software developers typically search online for reusable solutions to common programming problems. However, forming the question appropriately, and locating and integrating the best solution back to the code can be tricky and time consuming. As a result, several mining systems have been proposed to aid developers in the task of locating reusable snippets and integrating them into their source code. Most of these systems, however, do not model the semantics of the snippets in the context of source code provided. In this work, we propose a snippet mining system, named StackSearch, that extracts semantic information from Stack Overlow posts and recommends useful and in-context snippets to the developer. Using a hybrid language model that combines Tf-Idf and fastText, our system effectively understands the meaning of the given query and retrieves semantically similar posts. Moreover, the results are accompanied with useful metadata using a named entity recognition technique. Upon evaluating our system in a set of common programming queries, in a dataset based on post links, and against a similar tool, we argue that our approach can be useful for recommending ready-to-use snippets to the developer.

### 1 Introduction

Lately, the widespread use of the Internet and the introduction of the open-source development initiative have given rise to a new way of developing software. Developers nowadays rely more than ever on online services in order to solve common problems arising during development, including e.g. developing a component, integrating an API, or even fixing a bug. This new reuse paradigm has been greatly supported by search engines, code hosting facilities, programming forums, and question-answering communities, such as Stack Overflow[1]. One could even argue that software today is built using reusable components, which are found in software libraries and are exposed via APIs.

[1] https://stackoverflow.com/

As a result, the challenge lies in properly integrating these APIs/components in order to support the required functionality. This process is typically performed via *snippets*, i.e. small code fragments that usually perform clearly defined tasks (e.g. reading a CSV file, connecting to a database, etc.). Given the vastness of data in the services outlined in the previous paragraph (e.g. Stack Oveflow alone has more than 18 million question posts[2]), locating the most suitable snippet to perform a task and integrating it to one's own source code can be hard. In this context, developers often have to leave the IDE, form a query in an online tool and navigate through several solutions before finding the most suitable one.

To this end, several systems have been proposed. Some of these systems focus on the *API usage mining* problem [5,9,13,14,17,18,27,30] and extract examples for specific library APIs, while others offer more generic *snippet mining* solutions [3,6,28,29] and further allow queries for common programming problems (e.g. how to read a file in Java). Both types of systems usually employ an indexing mechanism that allows developers to form a query and retrieve relevant snippets.

These systems, however, have important limitations. First of all, several of them do not allow queries in natural language and may require the developer to spend time in order to form a query in some specialized format. Secondly, most systems index only information extracted from source code, without accounting for the semantics that can be extracted from comments or even from the surrounding text in the context (web location) that each snippet is found. Furthermore, most tools employ some type of lexical (term frequency) indexing, thus not exploiting the benefits of embeddings that can lead to semantic-aware retrieval. Finally, the format and the presentation of the results is most of the time far from optimal. There are systems that return call sequences as opposed to ready-to-use snippets, while, even when snippets are retrieved, they are sometimes provided as-is without any additional information concerning their APIs.

In this paper, we design and develop StackSearch, a system that receives queries in natural language and employs an indexing mechanism on Stack Overflow data in order to retrieve useful snippets. The indexing mechanism takes advantage of all possible information about a snippet by extracting semantics from both the textual (title, tags, body) and the source code part of Stack Overflow posts. The information is extracted using lexical matching as well as embeddings in order to produce a hybrid model and retrieve the most useful results, even when taking into account the possible ambiguities of natural language. Finally, the snippets retrieved by StackSearch are accompanied by relevant labels that provide an interpretation of the semantics of the posts and the employed APIs.

### 2 Related Work

As already mentioned, we focus on snippet mining systems that recommend solutions to typical programming problems. Some of the first systems proposed in this area were Prospector [16] and PARSEWeb [25]. These systems focus on

[2] Source: https://data.stackexchange.com/

recommending snippets that form a path between a source object to a target object. For Prospector, these paths are called jungloids and the program flow is a jungloid graph. Though interesting, the system has a local database, which limits its applicability. PARSEWeb, on the other hand, uses the Google search engine and produces better results in most scenarios [25]). However, both systems have important limitations; they require the developer to know which API calls to use and further receive queries in a specialized format, and not in natural language.

Another popular category of systems in current research involves those focusing on the challenge of API usage mining, such as MAPO [30], UP-Miner [27] or PAM [9]. The problem is typically defined as extracting common usage patterns from client source code, i.e. source code that uses the relevant API. To do so, MAPO employs frequent sequence mining, while UP-Miner uses graphs and mines frequent closed API call paths. PAM, on the other hand, employs probabilistic machine learning to extract sequences that exhibit higher coverage of the API under analysis and are more diverse [9]. Though quite effective, these systems are actually limited to the API under analysis and cannot support more generic queries. Furthermore, they too do not accept queries in natural language, while their output is in the form of sequences, instead of ready-to-use snippets.

Similar conclusions can be drawn for API mining systems that output snippets. For example, APIMiner [17] performs code slicing in order to generate common API usage examples, while eXoaDocs [14] further performs semantic clustering (using the DECKARD code clone detection algorithm [11]) to group them according to their functionality. CLAMS [13] also clusters the snippets and further generates the most representative (medoid) snippet of each cluster using slicing and code summarization techniques. Another interesting approach is MUSE [18], which employs a novel ranking scheme for the recommended snippets based on metrics such as the ease of reuse, a metric computed by determining whether a snippet has custom object types, and thus requires external dependencies. As with the previous approaches, these systems are effective for mining API usage examples, however they do not generalize to the problem of receiving natural language queries and retrieving API-agnostic reusable solutions.

This more generic snippet mining scenario is supported by several contemporary systems. One such system is SnipMatch [29], which employs pattern-based code search to retrieve useful snippets. SnipMatch, however, relies on a local index that has to be updated from the developer. More advanced systems in this aspect usually connect to online search engines and process their results to extract and recommend snippets. For example, Blueprint [3] and CodeCatch [6] employ the Google search engine, while Bing Code Search [28] employs Bing. Due to the integration with strong engines, these systems tend to offer effective natural language understanding features and their results are adequate even in less common queries. However, the text surrounding the code is not parsed for semantic information, so the quality of the retrieved snippets is bound only to the semantics introduced by the search engines. Moreover, the agnostic web search that these systems perform may often be suboptimal compared to issuing the queries to a better focused question-answering service.

These limitations have led to more specialized tools that employ Stack Overflow in order to recommend snippets that are proposed by the community and are accompanied by useful metadata. One of the first such systems is Example Overflow [35], an online code search engine that uses Tf-Idf as a scoring mechanism and retrieves snippets relevant to the jQuery framework. Two other systems in this area, which are built as plugins of the Eclipse IDE, are Prompter [22] and Seahawk [21]. Prompter employs a sophisticated ranking mechanism based not only on the code of each snippet, but also on metadata, such as the score of the post or reputation of the user that posted it on Stack Overflow. Seahawk also uses similar metadata upon building a local index using Apache Solr[3]. The main limitation of the systems in this category is their reliance on term occurrence; the lack of more powerful semantics restricts the retrieved results to cases where the query terms appear as-is within the Stack Overflow posts.

Finally, there are certain research efforts towards semantic-aware snippet retrieval. SWIM [23], for instance, which is proposed by the research team behind Bing Code Search [28], uses a natural language to API mapper that computes the probability $Pr(t|Q)$ that an API $t$ appears as a result to a query $Q$. The system retrieves the most probable snippets and synthesizes them to produce valid and human-readable snippets. A limitation of SWIM, which was highlighted by Gu et al. [10], is that it follows the bag-of-words assumption, therefore it cannot distinguish among certain queries (e.g. "convert number to string" and "convert string to number). The authors instead propose DeepAPI [10], a system that defines snippet recommendation as a machine translation problem, where natural language is the source language and source code is the target language. DeepAPI employs a model with three recurrent neural networks (one for the text of the query as-is, one for the same text reversed, and one to combine them) that retrieves the most relevant API call sequence given a query. The system, however, is largely based on code comments, so its performance depends on whether there is sufficient documentation in the snippets of its index. A similar approach is followed by T2API [20], another Eclipple plugin that uses a graph-based translation approach to translate query text into API usages. This system, however, is also largely based on synthesizing API calls and does not focus on semantic retrieval. Finally, an even more recent system is CROKAGE [24], which employs embeddings and further expands the query with relevant API classes from Stack Overflow. The final results are ranked according to multiple factors, including their lexical and semantic similarity with the query and their similar API usage.

In conclusion, the systems analyzed in the above paragraphs have the limitations that were discussed also in the introduction of this work. Several of them are focused only on APIs without generalizing to common programming problems. And while there are certain systems that allow queries in natural language, most of them rely on term frequency indexing and do not incorporate semantics extracted by the context of the snippets. In this work, we design a hybrid system that employs both a lexical (term frequency) and a word embeddings model on Stack Overflow posts' data. Note that, compared to source code comments that

[3] https://lucene.apache.org/solr/

may be incomplete or sometimes even non-existent, the text of Stack Overflow posts is a more complete source of information as it is the outcome of the explanation efforts of different members of the community [7]. As a result, our system can extract the semantic meaning of natural language queries and retrieve useful snippets, which are accompanied by semantic-aware labels.

## 3 StackSearch: A Semantic-aware Snippet Recommender

### 3.1 Overview

The architecture of StackSearch is shown in Figure 1. The left part of the figure refers to building the index while the right one refers to answering user queries.

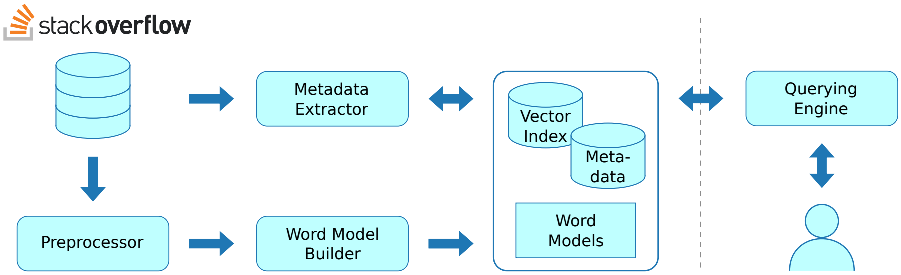

**Fig. 1.** Architecture of StackSearch

At first, our system retrieves information from Stack Overflow[4] and builds an SQLite[5] database of all Java posts. Note that our methodology is mostly language agnostic, however we use Java here as a proof of concept[6]. We created four tables in order to store question posts, answer posts, comments, and post links (to be used for evaluation, see subsection 4.2). For each of these tables we kept all information, i.e. title, tags, body, score, etc., as well as all connections of the data dump as foreign keys (e.g. any answer has a foreign key towards the corresponding question), so that we fully take into account the post context.

Upon storing the data in a suitable format, the Preprocessor receives as input all question posts, answer posts, and comments and extracts a corpus of texts. The corpus is then given to the Word Model Builder, which trains different models to transform the text to vector form. Finally, the system includes a vector index, where each set of vectors corresponds to to the title, tags, and body of one question post, the produced word models, and certain metadata for each question, which are extracted by the Metadata Extractor.

[4] We used the latest official data dump provided by Stack Overflow, which is available at https://archive.org/details/stackexchange

[5] https://www.sqlite.org/

[6] Applying our methodology to a different language requires only providing a preprocessor in order to extract the relevant source code elements from the post snippets.

When the developer issues a query, the Querying Engine initially extracts a vector for the query given the stored vector models, and then computes the similarity between the query vector and each vector in the vector index. The engine then ranks the results and presents them to the user along with their metadata. The steps required to build the index as well as the issuing of queries are discussed in detail in the following subsections.

### 3.2 Preprocessor

Upon creating our database, the next step is to preprocess the data in order to build the corpus that will be used to train our models. We extract the text and the code of each post by parsing the <pre> and <code> tags. We further remove all html tags from text and then perform a series of preprocessing steps. At first, the code is parsed to extract its semantic information. The posts are then filtered to remove the ones that introduce noise to the dataset and, finally, the texts are tokenized. These steps are outlined in the following paragraphs.

**Extracting Semantics from Source Code** Upon extracting the code from each question post, we parse it using an extension of the parser described in [8]. The parser checks if the snippets are compilable and also drops any snippets that are not written in Java. Upon making these checks, our parser extracts the AST of each snippet and takes two passes over it, one to extract type declarations, and one to extract method invocations (i.e. API calls). For example, in the snippet of Figure 2, the parser initially extracts the declarations is: InputStream, br: BufferedReader, and sb: StringBuilder (strings and exceptions are excluded). After that, it extracts the relevant API calls, which are highlighted in Figure 2.

```
// initialize an InputStream
InputStream is = new ByteArrayInputStream("sample".getBytes());
// convert InputStream to String
BufferedReader br = null;
StringBuilder sb = new StringBuilder();
String line;
try {
    br = new BufferedReader(new InputStreamReader(is));
    while ((line = br.readLine()) != null) { sb.append(line); }
} catch (IOException e) {
    e.printStackTrace();
} finally {
    if (br != null) {
        try { br.close(); } catch (IOException e) { e.printStackTrace(); }
    }
}
```

**Fig. 2.** Example snippet for “How to read a file line by line” (API calls highlighted)

Finally, the calling object of each API call is replaced by its type and the text of comments is also retrieved to produce the sequence shown in Figure 3.

initialize an InputStream, InputStream, ByteArrayInputStream, convert InputStream to String, BufferedReader, StringBuilder, StringBuilder, BufferedReader, InputStreamReader, BufferedReader.readLine, StringBuilder.append, BufferedReader.close

**Fig. 3.** Extracted sequence for the snippet of Figure 2

**Filtering the Posts** Filtering is performed using a classifier that rules out any posts that are considered by our system as noise. We used the regional CNN-LSTM model of Wang et al. [26], a model shown in Figure 4 that combines the CNN and LSTM architectures and achieves in capturing the characteristics of text considering also its order. Our classifier is binary; it receives as input the data of each post and its output determines whether a post is *useful* or *noisy*.

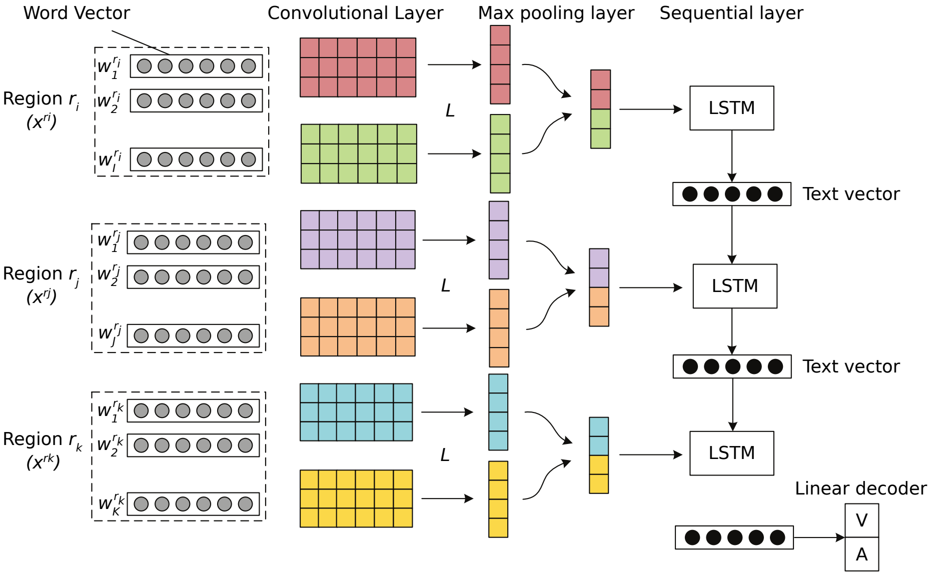

**Fig. 4.** Architecture of Regional CNN-LSTM model by Wang et al. [26]

The input embedding layer receives a one-hot encoding that corresponds to the concatenation of the title, body and tags of each post. Tokenization and one-hot encoding are performed before the text is given as input so no rules are given other than splitting on spaces and punctuation (this tokenization process is only used here on-the-fly to filter the posts, while we fully tokenize the text afterwards as described in the next paragraph). Punctuation marks are also kept as each of them is actually a token. After that, the classifier includes a CNN layer, which extracts and amplifies the terms (including punctuation) that cause noise. The CNN layer is followed by a max pooling layer that is used to reduce the number of parameters that have to be optimized by the model. Finally, the next layer is the LSTM that captures semantic information from nearby terms, which is finally given to the output to provide the binary decision.

To train our classifier, we have annotated a set of 2500 posts. For each post, we consider it noisy if it has error logs, debug logs or stack traces. Though useful in other contexts, in our case these posts would skew our models, as they contain a lot of generic data. Furthermore we deem noisy any posts with large amounts of numeric data (usually in tables) and any posts with code snippets in languages other than Java. The training was performed with accuracy as the metric to optimize, while we also used dropout to avoid overfitting. Upon experimenting with different parameter values, we ended up using the Adagrad optimizer, while the dropout and recurrent dropout parameters were set to 0.6 and 0.05 respectively. Setting the embedding length to 35 and the number of epochs to 5 proved adequate, as our classifier achieved accuracy equal to 0.94.

**Text Tokenization** Upon filtering, we now have a set of texts that must be tokenized before they are given as input to the models. Since tokenization might split Java terms (e.g. method invocations), we excluded these from tokenizing using regular expressions. After that, we removed all URLs and all non-alphabetical characters (i.e. numbers and special symbols) and tokenized the text.

#### 3.3 Word Model Builder

We build two models for capturing the semantics of posts, a Tf-Idf model and a FastText embedding. These models are indicative of lexical matching and semantic matching, respectively. They will serve as baselines and at the same time be used to build a more powerful hybrid model (see subsection 3.5). Both models are executed three times, one for the titles of the question posts, one for their bodies, and one for their tags. As already mentioned the code snippets are replaced by their corresponding text sequences, so they now are textual parts of the bodies. The two models are analyzed in the following paragraphs.

**Tf-Idf Model** We employ a vector space model to represent the texts (titles or bodies or tags) as documents and the words/terms as dimensions. The vector representation for each document is extracted using Tf-Idf vectorizer. According to Tf-Idf, the weight (vector value) of each term $t$ in a document $d$ is defined as:

$$tfidf(t, d, D) = tf(t, d) \cdot idf(t, D) \quad (1)$$

where $tf(t, d)$ is the term frequency of term $t$ in document $d$ and refers to the number of occurrences of the term $t$ in the document (title, body or tag). Also,

$idf(t, D)$ is the inverse document frequency of term $t$ in the set of all documents $D$, and is used as a normalizing factor to indicate how common the term is in the corpus. In our implementation (we used scikit-learn), $idf(t, D)$ is equal to $1 + log((1 + |D|)/(1 + d_t))$, where $|d_t|$ is the number of documents containing the term $t$, i.e. the number of titles, bodies or tags that include the relevant term. Intuitively, very common terms (e.g. "Java" or "Exception") may act as noise for our dataset, as they could appear to semantically different posts.

**FastText Model** FastText is a neural language model proposed by Facebook's AI Research (FAIR) lab [2,12]. Practically, fastText is a shallow neural network that is trained in order to reconstruct linguistic contexts of words. In our case, we transform the terms of the documents in one-hot encoding format and give the documents as input to the network during the training step. The result, i.e. the output of the hidden layer, is actually a set of word vectors. So, in this case, the resulting model is one where terms are represented as vectors. Given proper parameters, these vectors should incorporate semantic information, so that our model will have learned from the context.

We used the official implementation of fastText[7], selected the skip-gram variation of the model and we also set it up to use n-grams of size 3, 4, 5, 6, and 7. Upon experimenting with the parameters of the model, we ended up building a model with 300 dimensions and training it for 25 epochs. We used the negative sampling cost function (with number of negative samples equal to 10) and set the learning rate to 0.025 and the window size (i.e. number of terms that are within the context of a word) to 10. Also, the sampling threshold was set to $10^{-6}$, while we also dropped any words with fewer than 5 occurrences. Upon extracting all word vectors, we create the vector of each document level (title, body or tags) by averaging over its word vectors.

Finally, the output of either of our two models is a set of vectors, one for the title, one for the body and one for the tags of each post. In the case of Tf-Idf the dimensions of the vector are equal to the total number of words, while in the case of fastText there are 300 dimensions. In both cases, the vectors are stored in a vector index, which also contains ids that point to the original posts.

#### 3.4 Metadata Extractor

As metadata, we extract the named entities of each post, i.e. useful terms that may help the developer understand the semantics behind each post. To do so, we build a Conditional Random Fields (CRF) classifier [15], which performs named entity recognition based on features extracted from the terms themselves and from their context (neighboring terms). The goal is to estimate the probability that a term belongs to one of the available categories. To create a feature set for each term, we initially use two models.

At first, we employ the Brown hierarchical clustering algorithm [4] to generate a binary representation of all terms in the corpus. The algorithm clusters all

[7] https://github.com/facebookresearch/fastText terms in a binary tree structure. An example fragment of such a tree is shown in Figure 5. The leaf nodes of the tree are all the terms, so by traversing the tree from the root to a leaf we are given a binary representation known as *bitstring* for the corresponding term. Semantically similar terms are expected to share more similar tree paths. For instance, in the fragment of Figure 5, the terms 'array' and 'table' have binary representations 00100000 and 00100001 respectively, which are quite similar, as is their semantic meaning. The terms 'collection' and 'list' are also similar, yet somewhat less, as their representations (001000010 and 001001 respectively) differ more.

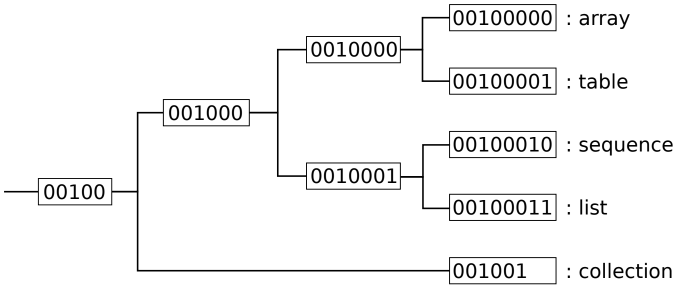

**Fig. 5.** Example Fragment of Binary Tree generated by the Brown Algorithm

Secondly, we use the fastText model of subsection 3.3. As already mentioned, our model extracts vector representations of terms so that semantically similar terms have vectors that are closer to each other (where proximity is computed using cosine similarity, see section 3.5). To reduce the size of these vectors (and thus avoid the curse of dimensionality), we further employ K-Means to cluster them into 5 configurations with different number of clusters (500, 1000, 1500, 2000, and 3000), an idea originating from similar natural language approaches [33, 34]. Thus, instead of using the term vector, we use 5 features for each term, each one corresponding to the id of the cluster that the feature is assigned.

Upon applying the two models, we finally build the feature set for the CRF classifier. Given each term $t_i$, its preceding term $t_{i-1}$ its following term $t_{i+1}$, we define their Brown bitstrings as $b_i$, $b_{i-1}$, and $b_{i+1}$ respectively, and we also define their K-Means cluster assignments as $k_i$, $k_{i-1}$, and $k_{i+1}$ respectively. Note that the $k_i$ includes all 5 cluster configurations used, thus producing on its own five features. Using these definitions, we build the following feature set:

– the term itself ($t_i$), and its combination with the preceding term ($t_{i-1}t_i$), and the following term ($t_i t_{i+1}$);
– the ids of the cluster assigned by K-Means to the term ($k_i$), the preceding term ($k_{i-1}$), and the following term ($k_{i+1}$);
– the bigram of the ids of K-Means clusters for the three terms ($k_{i-1}k_i k_{i+1}$);
– the bitstrings of the term ($b_i$), the preceding term ($b_{i-1}$), and the following term ($b_{i+1}$);

– the bigram of the three bitstrings ($b_{i-1}b_ib_{i+1}$);
– the prefixes with length 2, 4, 6, 8, 10, 12 of each one of the three bitstrings (e.g. for a bitstring 100100 the prefixes are 10, 1001, and 100100).

Finally, our features are augmented by employing the dataset proposed by Ye et al. [31,32]. The dataset comprises annotated entities extracted from Stack Overflow that lie in five categories: API calls, programming languages, platforms (e.g. Android), tools-frameworks (e.g. Maven), and standards (e.g. TCP). For each of these categories, we check whether the term is found in the corresponding dataset file and produce a true/false decision that is added as one more feature in our feature set. After that, we apply the CRF classifier for all terms and build a metadata index. Using this index we can produce a list of semantically rich named entities for each post in the dataset.

#### 3.5 Querying Engine

As already mentioned in subsection 3.3, the vector index comprises a set of vectors, three for each question post, corresponding to the title, the body and the tags of the post. When a developer issues a new query, it is initially preprocessed and tokenized, and then it is vectorized using either of our models. After that, we now have to produce a similarity score between each question post $p$ and the query $q$ of the developer. To do so, we use the following equation:

$$sim_{model}(q,p) = \frac{csim(v_q, v_{title(p)}) + csim(v_q, v_{body(p)}) + csim(v_q, v_{tags(p)})}{3} \quad (2)$$

where $v_q$ is the vector of the query and $v_{title(p)}$, $v_{body(p)}$, and $v_{tags(p)}$ are the vectors of the title, the body, and the tags of the question post respectively. Finally, $csim$ is the cosine similarity, which is computed for two vectors $v_1$ and $v_2$ as follows:

$$csim(v_1, v_2) = \frac{v_1 \cdot v_2}{|v_1| \cdot |v_2|} \quad (3)$$

Apart from the two models described so far, we also created a hybrid model by taking the average between the two scores computed by our models:

$$sim_{hybrid}(q,p) = \frac{sim_{Tf-Idf}(q,p) + sim_{fastText}(q,p)}{2} \quad (4)$$

This hybrid model incorporates the advantages of fastText, while giving more weight than only fastText to well-formed queries (i.e. with expected terms).

Finally, the user is presented with a list of possible results to the query, ranked according to their score. Each result contains information extracted by a question post and the corresponding answer posts. In specific, we include the title of the question post, the snippets extracted by the answer posts, the links to the question and answer posts (should the developer want to examine them), the Stack Overflow score of the answer posts, and the 8 most frequent named entities among all answer posts of the relevant question post. For example, assuming our system receives the query “How to read from text file?”, an example result is shown in Table 1. The developer can obviously select to check the second most relevant snippet of this question post, or even check another question post.

**Table 1.** Example StackSearch Response to Query "How to read from text file?"

| Type | Data |
|---|---|
| Post title | Reading a plain text file in Java |
| Question post link | https://stackoverflow.com/questions/4716503 |
| Top 8 labels | FileReader, BufferedReader, FileInputStream, InputStreamReader, Scanner.hasNext, Files.readAllBytes, FileUtils.readLines, Scanner |
| Snippet 1 | `Scanner in = new Scanner(new FileReader("file.txt"));`<br>`StringBuilder sb = new StringBuilder();`<br>`while(in.hasNext()) {`<br>`    sb.append(in.next());`<br>`}`<br>`in.close();`<br>`outString = sb.toString();` |
| Answer post link | https://stackoverflow.com/questions/4716556 |
| Answer post score | 117 |

### 4 Evaluation

To fully evaluate StackSearch, we perform three experiments. The first experiment involved annotating the results of common programming problems and is expected to illustrate the usefulness of our system. The second experiment relies on post links and is used to provide proof that our system is effective (and minimize possible threats to validity). Finally, for our third experiment, we compare StackSearch to the tool CROKAGE [24], which is quite similar to our system. Comparing StackSearch with other approaches was not possible, since several systems are not maintained and/or they are not publicly available (to facilitate researchers with similar challenges, we uploaded our code at https://github.com/AuthEceSoftEng/StackSearch).

#### 4.1 Evaluation using Programming Queries

We initially evaluate StackSearch using a set of common programming queries shown in Table 2. The dataset includes certain queries that are semantically very similar, which are marked as belonging to the same group, to determine whether our method captures the semantic features of the dataset. Queries in the same group call for the same solutions, i.e. their only difference is in the phrasing.

We evaluate all three implementations of our system, the Tf-Idf model, the fastText model, and the hybrid model. For each implementation, upon giving the queries as input, we retrieve the first 20 results and annotate them as relevant or non-relevant. A result is marked as relevant if its snippet covers the functionality that is described by the query. We gathered the results of all three algorithms together and randomly permuted them, so the annotation was performed without any prior knowledge about which result corresponds to each model, in order to be as objective as possible.

**Table 2.** Dataset used for Semantically Evaluating StackSearch

| ID | Query | Group |
|---|---|---|
| 1 | How to read a comma separated file? | 1 |
| 2 | How to read a CSV file? | 1 |
| 3 | How to read a delimited file? | 1 |
| 4 | How to read input from console? | 2 |
| 5 | How to read input from terminal? | 2 |
| 6 | How to read input from command prompt? | 2 |
| 7 | How to play an mp3 file? | 3 |
| 8 | How to play an audio file? | 3 |
| 9 | How to compare dates? | 4 |
| 10 | How to compare time strings? | 4 |
| 11 | How to dynamically load a class? | 5 |
| 12 | How to load a jar/class at runtime? | 5 |
| 13 | How to calculate checksums for files? | 6 |
| 14 | How to calculate MD5 checksum for files? | 6 |
| 15 | How to iterate through a hashmap? | 7 |
| 16 | How to loop over a hashmap? | 7 |
| 17 | How to split a string? | 8 |
| 18 | How to handle an exception? | 9 |

For each query, we evaluate each implementation by computing the average precision of the results. Given a ranked list of results, the average precision is computed by the following equation:

$$AveP = \frac{\sum_{k=1}^{n} (P(k) \cdot rel(k))}{number\ of\ relevant\ results} \quad (5)$$

where $P(k)$ is the precision at $k$ and corresponds to the percentage of relevant results in the first $k$, and $rel(k)$ denotes if the result in the position $k$ is relevant. We also use the mean average precision, defined us the mean of the average precision values of all queries.

We calculated the average precision at 10 and 20 results. The values for each query are shown in Figure 6. As shown in these graphs, the fastText and the hybrid models clearly outperform the Tf-Idf model, which is expected as they incorporate semantic information. We also note that the hybrid implementation is even more effective than fastText for most queries. Interestingly, there are certain queries in which Tf-Idf outperforms one or both of the other implementations. Consider, for example, query 17; this is a very specific query with clear terms (i.e. developers would rarely form such a query without using the term 'string') so there is not really any use for semantics. For most queries, however, better results are proposed by fastText or by our hybrid model.

We note, especially, what is the case with queries in the same group (divided by gray lines in the graphs of Figure 6). Given, for instance, the second group, query 4, which refers to input from the console, returns multiple useful results using any of the three models. The results, however are quite different for queries

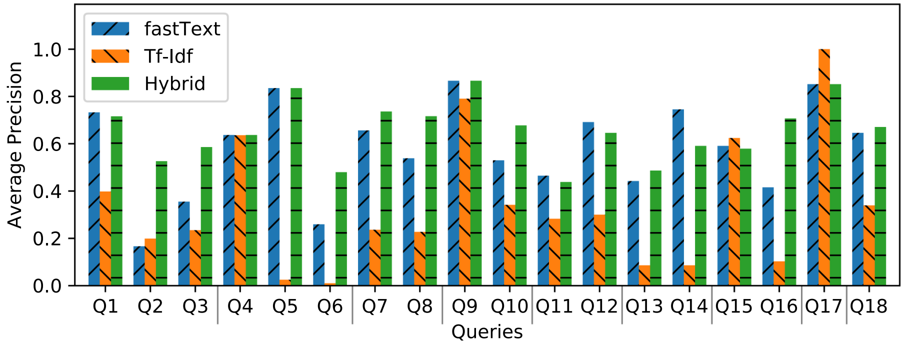

(a)

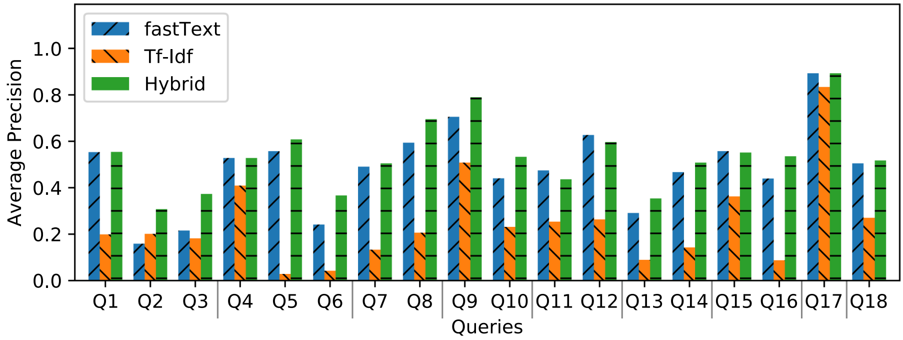

(b)

**Fig. 6.** Average Precision for the three Implementations (a) at 10, and (b) at 20 Results

5 and 6, which are similar albeit for the replacement of the term 'console' with 'terminal' and with 'command prompt' respectively. This indicates that our word embedding successfully captures the semantics of the text and considers the aforementioned terms as synonyms. This advantage of our system is also clear in group 1 (comma-separated vs CSV vs delimited file), group 4 (dates vs time strings), etc., and even in more difficult semantic relationships, such as the one of group 5 (i.e. loading dynamically vs at runtime).

Finally, we calculated the mean average precision for the same configurations as before. The values for the three implementations are shown in Figure 7a, where it is clear once again that the word embeddings outperform the Tf-Idf model, while our hybrid model is the most effective of the three models.

To further outline the differences among the models we also computed the mean search length. The search length is a very useful metric since it intuitively simulates the process used when searching for relevant results. The metric is defined as the number of non-relevant results that one must examine in order to find a number of relevant results. We computed the search length for all queries for finding from 1 up to 10 relevant results.

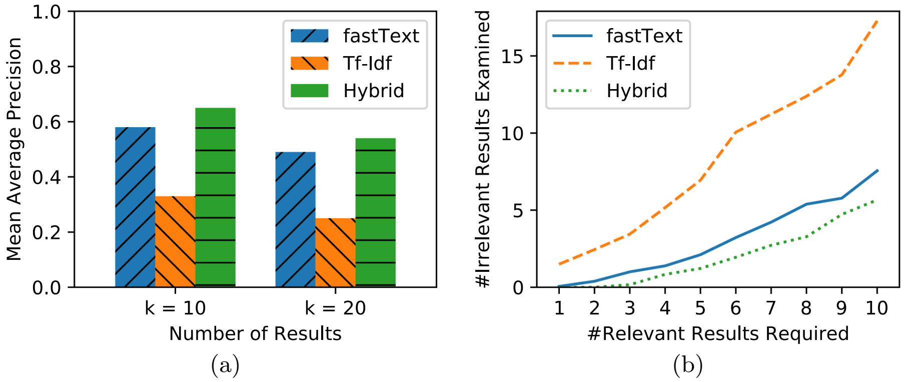

**Fig. 7.** Results depicting (a) the Mean Average Precision, and (b) the Mean Search Length, for the three Implementations

Averaging over all queries provides the mean search length, of which the results are shown in Figure 7b. The results are again encouraging for our proposed models. Indicatively, to find the first useful result, the developer has to examine less than 0.1 irrelevant results on average for fastText and for the hybrid model, whereas using Tf-Idf requires examining 1.5 irrelevant results. Furthermore, when the developer skims over the results of fastText, he/she will only need to view 2.11 irrelevant snippets on average, before finding the first 5 relevant. Using the hybrid model, he/she will only need to see 1.22. Tf-Idf is clearly outperformed in this case, providing on average almost 7 irrelevant results, along with the first 5 relevant. Similar conclusions can be drawn for the first 10 relevant results. In this case, the developer would need to examine around 17.5, 7.5, and 5.5 results on average, for Tf-Idf, fastText, and our hybrid model, respectively.

#### 4.2 Evaluation using Post Links

The main goal of the previous subsection was to illustrate the potential of our word embedding models. The results, especially for the groups of queries, have shown that our models indeed capture the semantics of text. As already mentioned, the annotation process was performed in such a way to limit any threats to validity. Nevertheless, to further strengthen the objectivity of the results, we perform one more experiment, which is described in this subsection.

In the lack of a third-party annotated Stack Overflow dataset, what we decided to do is evaluate our models using the post links provided by Stack Overflow, an idea found in [8]. In Stack Overflow, the presence of a link between two questions is an indicator that the two questions are similar. Note, of course, that the opposite assumption, i.e. that any two questions that are not linked are not similar to each other, is not necessarily correct. There are many questions that are asked and perhaps not linked to similar ones. In our evaluation, however, we formulate the problem as a search/retrieval scenario, so we only use post links to determine whether our models can retrieve objectively relevant results.

To create our link evaluation dataset, we first extracted all post links of Java question posts. After that, for performance reasons, we dropped any posts without snippets and any posts with Stack Overflow score lower or equal to -3, as these are not within the scenario of a system that retrieves useful snippets. These criteria reduced the number of question posts to roughly 200000 (as opposed to the original dataset that had approximately 1.3 million question posts). These question posts have approximately 37000 links, reinforcing our assumption that non-linked questions are not necessarily dissimilar.

We execute StackSearch with all three models giving as queries the titles of all question posts of the dataset. For each query, we retrieve the first 20 results (as we may assume this is the maximum a developer would normally examine). We determine how many of these 20 results are linked to the specific question post, and compute the percentage of relevant results compared to the total number of relevant post links of the question post. By averaging over all queries (i.e. titles of question posts of the dataset), we compute the percentage of relevant links retrieved on average for each model. The results are shown in Figure 8.

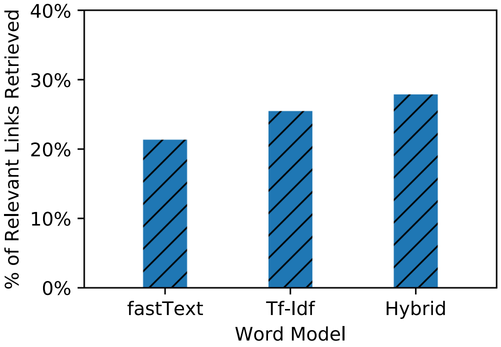

**Fig. 8.** Percentage of Relevant Results (compared to the number of Links of each Question Post) in the first 20 Results for the three Implementations

At first, one may note that the results for all models are below 30%, a rather low number, which is however expected, given the shortcomings of our dataset. Many retrieved results are actually relevant, however they are not linked to the question posts of the queries. In any case, we are given an objective relative comparison of the three models. And this comparison provides some interesting insights. An interesting observation is that Tf-Idf outperforms fastText. This is not totally unexpected, if we consider that the post links of Stack Overflow are created by the community, therefore it is possible that posts with similar meanings but different key terms are not linked. As a result, fastText may discover several posts that should be linked, yet they are not. On the other hand, Tf-Idf focuses on identical terms which are rather easier to discover using the Stack Overflow service. In any case, however, our hybrid model outperforms Tf-Idf and fastText, as it combines the advantages of Tf-Idf and fastText.

#### 4.3 Comparative Evaluation

Upon demonstrating the effectiveness of StackSearch in the previous subsections, we now proceed to compare it with a similar system, the tool CROKAGE. To do so, we have employed the dataset proposed by CROKAGE [24]. The dataset involves 48 programming queries, similar to those introduced in subsection 4.1. The queries include diverse tasks, such as comparing dates, resizing images, pausing the current thread, etc.

Given that our dataset comprises Stack Overflow posts, it can be used to assess both tools. Thus, we issued the queries at both StackSearch and CROKAGE. The results of the queries had been originally annotated by two annotators (of which the results were merged) in Stack Overflow posts, marking any post as relevant if it addresses the query with a feasible amount of changes [24]. So we have used these annotations and only had to update a small part of them in order to make sure that they are on par with our dataset, which includes the latest data dump of Stack Overflow. As before, for each query we have calculated the average precision at 5 and 10 results as well as the search length for finding 1 up to 10 relevant results. The mean average precision and the mean search length results for the two tools are shown in Figures 9a and 9b, respectively (results per query are omitted due to space limitations).

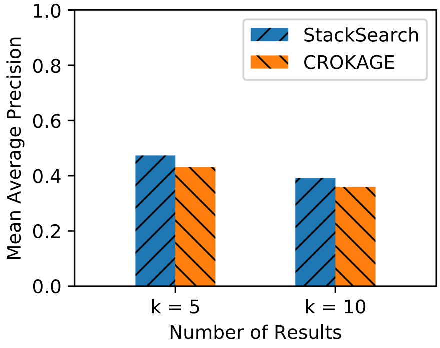

(a)

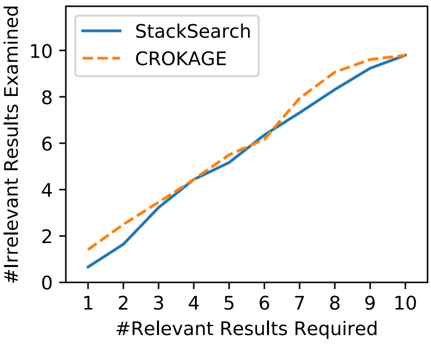

(b)

**Fig. 9.** Results depicting (a) the Mean Average Precision, and (b) the Mean Search Length, for StackSearch and CROKAGE

Both tools seem to be effective on the provided dataset. Concerning mean average precision, StackSearch outperforms CROKAGE both at 5 and at 10

results, indicating that it retrieves more useful results on average. Moreover, it seems that their difference is more noticeable when a fewer number of results is required, indicating that StackSearch provides a better ranking.

This difference is also illustrated by the mean search length for the two approaches. Indicatively, using StackSearch, the developer will need to examine only 0.66 irrelevant snippets on average, before finding the first relevant one (the corresponding value for CROKAGE is 1.42). Our tool also performs better for finding the second and third relevant results, while the two tools perform equally well for finding five or more results.

### 5 Conclusion

Although several API usage and snippet mining solutions have been proposed, most of them do not account for the semantics of the source code and the surrounding text. Furthermore, most contemporary systems do not employ word embeddings to enable semantic-aware retrieval of snippets, and are limited either by the format of their input, which is not natural language, or by their output, which is not ready-to-use snippets. In this work, we have created a novel snippet mining system that extracts snippets from Stack Overflow and employs word embeddings to model code and as well as contextual information. Given our evaluation, we conclude that the hybrid model of StackSearch effectively extracts the semantics of the data and outperforms both our baselines (Tf-Idf and fastText) as well as the snippet mining tool CROKAGE. Finally, our system accompanies the retrieved snippets with useful metadata that convey the meaning of each post.

Future work lies in several directions. At first, we may employ a more sophisticated ranking scheme using more information from Stack Overflow (e.g. the Stack Overflow score of the snippet's answer post) or even from other sources (e.g. the reuse rate of Stack Overflow snippets in GitHub [1]) and assess the influence of that information on the effectiveness of the scheme. Furthermore, we could employ different word embedding techniques or even variations of fastText, such as the combination of the In-Out vectors of fastText [19]. We could also further investigate our hybrid solution, implementing a more complex scheme other than averaging the scores of the two models. Finally, we could further assess StackSearch using a survey to ask developers whether the system actually retrieves useful snippets and whether it reduces the effort required for finding and integrating reusable snippets.

### Acknowledgements

This research has been co-financed by the European Regional Development Fund of the European Union and Greek national funds through the Operational Program Competitiveness, Entrepreneurship and Innovation, under the call RESEARCH - CREATE - INNOVATE (project code: T1EDK-02347).
